# Early life exposure to unpredictable parental sensory signals shapes cognitive development across three species

**DOI:** 10.3389/fnbeh.2022.960262

**Published:** 2022-10-20

**Authors:** Elysia Poggi Davis, Kai McCormack, Hina Arora, Desiree Sharpe, Annabel K. Short, Jocelyne Bachevalier, Laura M. Glynn, Curt A. Sandman, Hal S. Stern, Mar Sanchez, Tallie Z. Baram

**Affiliations:** ^1^Department of Psychology, University of Denver, Denver, CO, United States; ^2^Department of Pediatrics, University of California, Irvine, Irvine, CA, United States; ^3^Department of Psychology, Spelman College, Atlanta, GA, United States; ^4^Emory National Primate Research Center, Emory University, Atlanta, GA, United States; ^5^Department of Statistics, University of California, Irvine, Irvine, CA, United States; ^6^Mary Frances Early College of Education (MFECOE) Torrance Center for Creativity and Talent Development, University of Georgia, Athens, GA, United States; ^7^Department of Psychology, Chapman University, Orange, CA, United States; ^8^Department of Psychiatry and Human Behavior, University of California, Irvine, Irvine, CA, United States; ^9^Department of Psychiatry and Behavioral Sciences, School of Medicine, Emory University, Atlanta, GA, United States; ^10^Department of Anatomy and Neurobiology, University of California, Irvine, Irvine, CA, United States; ^11^Department of Neurology, University of California, Irvine, Irvine, CA, United States

**Keywords:** unpredictability, stress, early adversity, memory, development, cognition, parental care, monkeys

## Abstract

Exposure to early life adversity has long term consequences on cognitive function. Most research has focused on understanding components of early life adversities that contribute to later risk, including poverty, trauma, maltreatment, and neglect. Whereas these factors, in the aggregate, explain a significant proportion of emotional and cognitive problems, there are serious gaps in our ability to identify potential mechanisms by which early life adversities might promote vulnerability or resilience. Here we discuss early life exposure to unpredictable signals from the caretaker as an understudied type of adversity that is amenable to prevention and intervention. We employ a translational approach to discover underlying neurobiological mechanisms by which early life exposure to unpredictable signals sculpts the developing brain. First, we review evidence that exposure to unpredictable signals from the parent during sensitive periods impacts development of neural circuits. Second, we describe a method for characterizing early life patterns of sensory signals across species. Third, we present published and original data illustrating that patterns of maternal care predict memory function in humans, non-human primates, and rodents. Finally, implications are discussed for identifying individuals at risk so that early preventive-intervention can be provided.

## I Unpredictable parental signals and neural circuit development

Cognitive health and vulnerabilities involve an interplay of genes and environment, especially during sensitive developmental periods. Cognitive functions are a result of the development and maturation of underlying brain circuits. These circuits, built of neurons and neuronal ensembles, are connected *via* synapses, and perform the complex computational tasks underlying specific brain functions. During early life, immature circuits are sculpted as certain synaptic connections are strengthened because of their activation, and others are eliminated. For sensory circuits supporting vision and hearing, it is established that patterns of sensory signals are required for their maturation (Wiesel and Hubel, 1963; Khazipov et al., 2004; Hackett et al., 2011; Espinosa and Stryker, 2012; Singh-Taylor et al., 2018a; Takesian et al., 2018). Light patterns for example, drive normative maturation of circuits within the visual system and disruption of these patterns leads to deficits in visual function (Espinosa and Stryker, 2012). Further, patterns of maternal auditory signals in maternal speech have been shown to affect functional connectivity in neonatal language neurocircuits (Uchida-Ota et al., 2019). These studies establish the importance of patterns of sensory information for neural circuit formation (Davis et al., 2017; Birnie and Baram, 2022). During the sensitive period of infancy, sensory signals come predominantly from the parent and as documented by classic work in non-human primates (Harlow, 1964; Harlow et al., 1965) and rodents (Denenberg and Karas, 1959; Denenberg and Bell, 1960; Levinek, 1967; Hofer, 1970, 1973; Hennessy et al., 1980), parental sensory signals are critical for infant development. Across species, the enduring impact of quality of parental care (e.g., sensitivity, responsiveness) is known (Bowlby, 1950; Ainsworth et al., 1978; Feldman, 2007, 2015; Landers and Sullivan, 2012; Beebe et al., 2016; Howell et al., 2017). However, much less is understood regarding the role of sequences and patterns of parental sensory signals during sensitive periods. Based on evidence supporting the importance of patterns of sensory signals for sensory circuit formation and the primacy of parental signals, evaluation of the impact of patterns of parental sensory signals on higher order circuit development, such as those involved in memory, reward, or stress, is warranted (Baram et al., 2012; Davis et al., 2017; Glynn and Baram, 2019; Noroña-Zhou et al., 2020; Birnie and Baram, 2022).

Infants are exposed to a variety of sensory information (i.e., auditory, tactile, and visual), primarily from their parents. The information may be patterned or sequential (e.g., a parent may regularly follow an auditory signal with simultaneous auditory and visual signals), or it may be inconsistent and without order (e.g., a parent may follow an auditory signal with various unpredictable sensory signals). We have developed an approach to quantify unpredictability across species by computing entropy rates. These define the degree to which one can deduce the next parental behavior from the most recent behavior, providing an index of the predictability of sensory signals from mother to her infant/pup. We have reported that the predictability of sensory signals early in life, characterized by entropy, associates with cognitive and emotional outcomes later in development (Davis et al., 2017; Glynn et al., 2018).

Emerging evidence demonstrates that early exposure to unpredictable patterns of maternal signals (high entropy) correlates with mental development (Davis et al., 2017) and stress regulation, (Noroña-Zhou et al., 2020) as well as the ability to regulate behavior, cognition, and emotion in children from 1 to 9 years of age (Davis et al., 2019). The links between exposure to unpredictable signals and later outcomes were observed in independent cohorts, from California, USA and Turku, Finland with very different backgrounds and cultures, and persisted after accounting for parental mental health, socioeconomic status, and maternal sensitivity, indicating the robustness of the links with unpredictability (Davis et al., 2019). Further, sequences of parental signals are predictive of the maturation of child brain circuits. Unpredictability early in life altered the balanced development of two temporo-frontal projections, the uncinate fasciculus and the cingulum, assessed with diffusion tensor imaging (DTI), and aberrant development of these two regions contributed to performance on a memory task. Specifically, imbalance between the uncinate fasciculus and the cingulum partially mediated the association between exposure to unpredictable signals during infancy and poor memory performance on a delayed object recognition task during childhood (Granger et al., 2021). Importantly, these effects of unpredictable parental signals on brain and behavior are independent of key previously established predictors of development including sensitivity and responsiveness of parental care, parental mental health, and socioeconomic factors (e.g., income, education, etc.), underscoring the importance of patterns of unpredictability in shaping the immature brain.

Controlled mouse and rat studies bolster human research by illustrating the causal role of unpredictability in shaping subsequent outcomes. In such preclinical studies, unpredictable patterns of dam behaviors during interactions with the pup directly led to aberrant memory and emotional circuit maturation and function in the offspring (Molet et al., 2016a; Bolton et al., 2018, 2020, 2022). For example, in the hypothalamus, a key node of the stress circuit, unpredictable patterns of maternal care behaviors influence synaptic connectivity (Korosi et al., 2010; Gunn et al., 2013; Singh-Taylor et al., 2018b) by attenuating microglial synaptic pruning (Bolton et al., 2022). Further, the same unpredictable maternal behavior sequences led to impoverished apical dendritic trees in hippocampal pyramidal cells and such disrupted hippocampal neuronal structure are associated with memory disfunction (Molet et al., 2016a). These experimental findings underscore the role of unpredictable patterns of parental signals on shaping neural circuit development.

## II Characterizing unpredictable parental signals across species

Growing evidence supports the importance of patterns of parental sensory signals on brain development across species. We have applied the same basic approach of characterizing unpredictability is used across species, with the major differences having to do with the specific sensory signals that are assessed and the appropriate developmental epoch for the assessments. Entropy is a concept associated with randomness that arises throughout science from the study of heat and other forms of energy, where it was initially introduced to the study of the mechanics of interacting particles and the transmission of information. We apply it in this latter sense, using definitions provided by Shannon (Vegetabile et al., 2019) to characterize the predictability of the next word or symbol to be transmitted over a communication channel. The entropy rate of a sequence, of parental behaviors or signals in our case, is a quantitative measure of the randomness or unpredictability of the next observation in the sequence. Below we provide details for computation of entropy rate to characterize unpredictability and we then, using the example of memory, illustrate the way that unpredictability impacts cognitive function in humans, monkeys, and mice.

### A. Unpredictable parental signals: Humans

**FIGURE 1:**
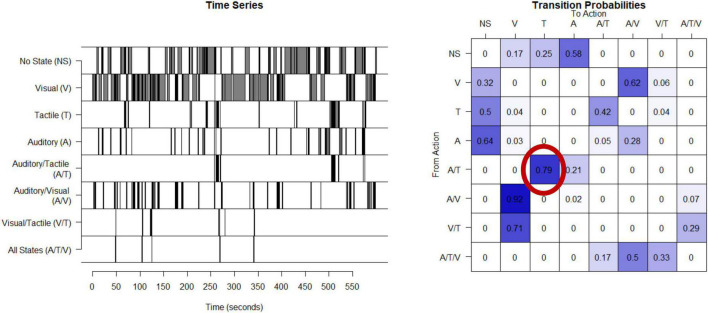
Calculation of entropy rate in humans: This time series shows the eight possible states for one example mother given the three types of sensory signals. This timeseries is then used to compute a transitional probability matrix. This matrix shows the percent of times a mother transitions from one state to another. For example, as shown in the probability matrix, 79% of the time when this mother was providing auditory and tactile at the same time, she then transitions to providing only tactile.

Unpredictability of sensory inputs (visual, tactile, and auditory) are derived from observations of mothers interacting with their children in a semi-structured 10-min play episode (NICHD, 2001). During these play interactions, mothers are given a standard set of age-appropriate toys. Maternal behaviors which provide auditory (A; e.g., any speech or laughter), visual (V; e.g., showing a toy while the child is attending to the toy), or tactile (T; e.g., touching or holding the child) signals to the child are coded continuously in real time by coders who are blinded to all other participant characteristics with 20% of the videos coded independently for interrater reliability. Details of the coding scheme are available in Davis et al. (2017) and at https://contecenter.uci.edu/. From the 3 coded types of sensory signals (auditory, tactile and visual) there are 8 categories of sensory input the mother can provide at any point in time; these include only auditory, tactile, or visual stimuli, any combination of two (e.g., auditory and tactile), all three of these signals (auditory, tactile and visual) at the same time, or no input. The timeseries of each mother’s signals, example shown in Figure 1, is used to create a matrix of transition probabilities, with each entry in the matrix capturing the proportion of the time that the mother transitions from providing the signal identified by the row (e.g., visual) to providing the signal identified by the column (e.g., auditory and visual at the same time) (Figure 1). An entropy rate is then calculated using the approach described in the appendix with R code available.^1^ The entropy rate of the process (as defined, e.g., in Cover and Thomas (2006)) measures the randomness and unpredictability of the distribution of transitions with higher values indicating less predictable maternal signals. Computation and applications of the entropy rate estimates are described in more detail in Vegetabile et al. (2019) and in the Supplementary material. The entropy rate is stable within a given mother-child play session (entropy derived from the first and second half of a 10-min play period have correlation 0.5) and across sessions from when the infant is 6–12 months of age (Vegetabile et al., 2019).

### B. Unpredictable parental signals: Non-human primates

**FIGURE 2:**
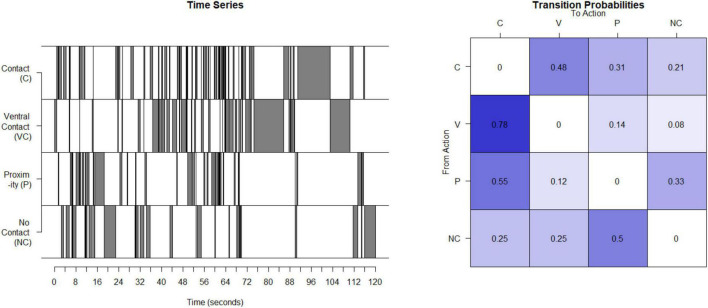
Calculation of entropy rate in monkeys: This time series illustrates the four possible states for one example mother. This timeseries is then used to compute a transitional probability matrix. This matrix shows the percent of times a mother transitions from one state to another.

Unpredictable maternal signals in the monkeys were assessed using a process very similar to our work with humans. Interactions of mother and infant were observed for 4 days in the third month post birth for 30 min per day. Based on developmental age, 3 months in the monkey is most analogous to the data from 12-month-old infants in humans and was the data used here (Mattison and Vaughan, 2017). The maternal behaviors identified and recorded focus on the presence and type of contact between the mother and their infant. Coded maternal behaviors that provide signals to the infant are contact (contact other than ventral) (C), ventral contact (V), proximity to the infant (P), and not in contact at all (NC). Sequences of the 4 possible contact-related behaviors were recorded for each period, concatenated to provide a time series (see Figure 2), the matrix of transition probabilities calculated (see Figure 2), and the entropy rate computed as described above for humans.

### C. Unpredictable parental signals: Rodents

**FIGURE 3:**
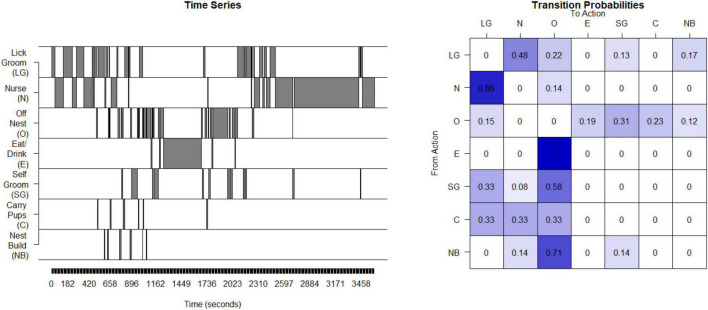
Calculation of entropy rate in rodents: This time series of the seven possible states for one example litter in rodents (mice). This timeseries is then used to compute a transitional probability matrix. This matrix shows the percent of times a mother transitions from one state to another.

Unpredictability of maternal behavior for rats and mice was assessed using the same basic approach as for the humans and non-human primates. Interactions between the dam and her pups were observed during postnatal days 2 through 7 for 250-min periods per day. The maternal behaviors that are the source of sensory signals to the pups were nursing (N), licking and grooming (LG), carrying pups (C), eating/drinking (E), off the nest (O), nest-building (NB) and self-grooming (SG) (Molet et al., 2016b). The data from the observation periods were concatenated into a single time series (see Figure 3). The matrix of transition probabilities was calculated (see Figure 3) and the entropy rate was computed as described above for humans.

## III Unpredictable parental signals and cognition cross-species

The approach we have described to characterizing unpredictable parental signals using entropy rate provides a novel technique that can be used to test the impact of patterns of moment-to moment parental signals on the developing brain. Leveraging research with human infants and children highlighting the importance of patterns of moment-to-moment signals and combining this research with translational studies in non-human primates and rodents provides: (1) support for evolutionarily-conserved processes that link patterns of maternal signals to infants and children’s development of learning/memory and emotional regulation brain systems, and (2) mechanistic insight into novel processes by which sequences of parental signals shape the organization of the developing brain. This approach provides an opportunity to identify systems that are susceptible to exposure to early life unpredictability. We test here the link between unpredictable patterns of sensory signals from the parent to the infant on memory development, in humans, monkeys, rats and mice.

### A. Unpredictable parental signals and memory function: Humans

We have previously shown that exposure to unpredictable maternal signals during infancy (high entropy rate at 6 and 12 months) predicted poorer recall memory, (Davis et al., 2017) assessed using a delayed recall memory task (Sheslow and Adams, 2003) that is indicative of hippocampal function (Squire et al., 2007) at 6.5 years of age. Associations with unpredictability persisted after covarying for maternal depression symptoms, maternal sensitivity, and socioeconomic status. Further, unpredictable patterns of parental signals rather than counts of behaviors or counts of transitions predicted child cognitive function, suggesting that patterns of signals rather than simply the number of signals is important in shaping later outcomes (Davis et al., 2017). Notably, this study provided parallel evidence from a preclinical experimental model that early life exposure to unpredictable signals causes poor memory performance on an object recognition task among adolescent rats, (Davis et al., 2017) supporting the likelihood that unpredictable signals underlie the observed associations in humans.

**FIGURE 4:**
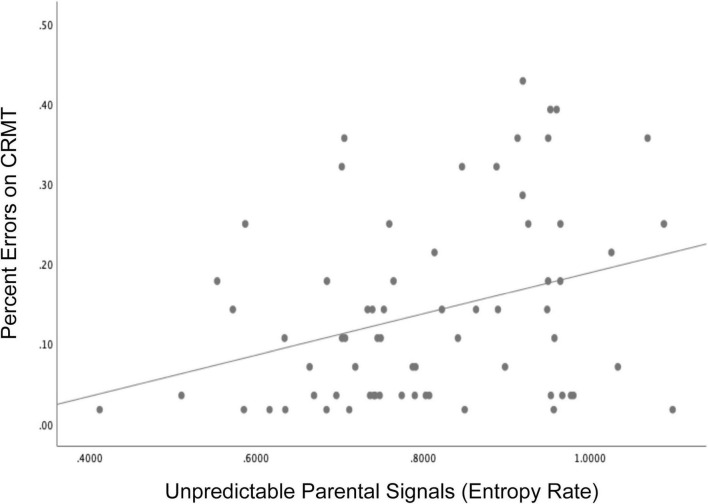
Children who were exposed to more unpredictable parental signals (higher entropy rate) during infancy (6 and 12 months) showed poorer memory performance indicated by more errors on the Continuous Recognition Memory task (CRMT) at 6.5 years of age, *r* = –0.316, *p* < 0.01.

Consistent with these published findings, we present here new findings linking exposure to unpredictable sensory signals in infancy to child memory performance in 71 mothers and their children (41 girls) participating in a larger longitudinal study of early life experiences and development (Glynn et al., 2018). Initial recruitment criteria included: (1) singleton pregnancy, (2) over the age of 18, (3) English speaking, (4) non-smoking. Participants in the current study additionally participated in infant assessments of maternal child behavior. Children were 34% Hispanic/Latinx, 41% white, 3% Black, and 18% multiracial/ethnic and lived in households with an average income to needs ratio of 3.2. The Institutional Review Board at the University of California, Irvine reviewed, and approved study protocols and mothers gave written and informed consent for themselves and their children. Unpredictability of sensory signals were evaluated when the child was 6 and 12 months and entropy rate was computed as described in section II (Figure 1). The Continuous Recognition Memory task (CRMT), (Sher, 2006) an object recognition memory task that has previously been shown to engage medial temporal regions such as the hippocampus (Eichenbaum et al., 2007) was administered at 6.5 years (see Supplementary Figure 1 for task details). Consistent with published findings, exposure to unpredictable parental sensory signals during infancy (high entropy rate) was associated with poorer memory as indicated by more errors (lower accuracy) on the CRMT at 6.5 years of age (Figure 4, *r* = −0.316, *p* < 0.01). Associations remained after covarying socio-demographic factors and assessments of maternal sensitivity, β = −0.27, *t* = −2.09, *p* = 0.04. These findings underscore the importance of patterns of unpredictability in shaping the immature brain and consistent with published research suggesting that memory functions may be susceptible to early life unpredictability (Heidinger et al., 2012; Molet et al., 2016a; Davis et al., 2017).

### B. Unpredictable parental signals and memory function: Non-human primates

We sought additional evidence to test the idea that the impact of unpredictable signals is an evolutionarily conserved processes by assessing the biological importance of unpredictable patterns of maternal sensory signals and memory development with non-human primates. Specifically, we conducted an evaluation of links between unpredictable patterns of parental signals and memory performance in juvenile rhesus monkeys, assessed at a similar developmental stage as the children in the studies described above. Unpredictability of maternal behaviors was assessed in 21 mother- infant dyads, rhesus macaques -*Macaca mulatta* who were part of a larger longitudinal developmental study (McCormack et al., 2015; Drury et al., 2016; Howell et al., 2019; Morin et al., 2020). Nine of the infants in this study experienced maltreatment by their mothers (3 females) and 12 were non-maltreated/control infants (6 females). All animals were socially-housed at the Emory National Primate Research Center (ENPRC), with social dominance status (high, medium, low) counterbalanced across groups and ≥450 grams birth weight to exclude prematurity. All procedures were approved by the Emory Institutional Animal Care and Use Committee and performed in accordance with the NIH Guide for the Care and Use of Laboratory Animals.

**FIGURE 5:**
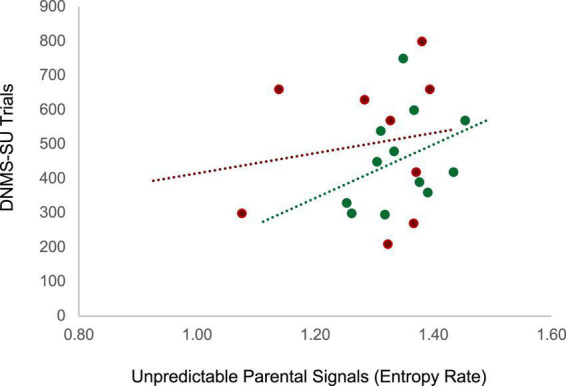
Juvenile monkeys who experienced more maternal unpredictable signals (higher entropy rate) during infancy (3 months) showed impaired working memory performance as indicated by the higher number of trials to reach criterion on the Delayed non-matching to sample -session unique (DNMS-SU) task as juveniles (18–24 months). Green dots represent animals in the control group (*r*(12) = 0.37, *p* = 0.12) and red dots represent animals in the maltreatment group (*r*(9) = 0.16, *p* = 0.34).

Unpredictability was characterized by coding maternal parenting behaviors when the infant was 3 months of age and entropy rate was computed as described in section II (see Figure 2). Working memory in the juvenile animals was assessed at 18 months of age, roughly equivalent to 5–6 year old children, using the delayed non-matching to sample task, task trial unique (DNMS-TU), a simple recognition memory task, followed by the DNMS-session unique (DNMS-SU), which is a more complex working memory task (Heuer and Bachevalier, 2011) (see Supplementary material andSupplementary Figure 2 for task details). Juvenile monkeys who were exposed to more unpredictable maternal signals during infancy (higher entropy rate) showed impaired working memory performance as indicated by the higher number of trials to reach criterion on the DNMS-SU task. For the control animals, experiencing unpredictable maternal signals during infancy (higher entropy rate) was associated with poorer performance (more trials to criterion) on the DNMS-SU task, *r*(12) = 0.37, *p* = 0.12 (see Figure 5). Associations were weaker among the animals who experienced maltreatment, *r*(9) = 0.16, *p* = 0.34. Although underpowered to reach statistical significance, the magnitude of the effect size is consistent with analogous studies with humans. These preliminary results require replication in future research. Together, human and monkey studies provide suggestive evidence that unpredictable patterns of sensory signals during early infancy are a potent signal shaping brain circuit maturation across species.

### C. Unpredictable parental signals and memory function: Rodent models

While the existing human and monkey work provide evidence that links between unpredictability and memory are conserved across species, these observational projects do not allow tests of causality. This limitation is addressed in experimental studies of early life unpredictability and several types of memory in juvenile and adult rats and mice. Earlier rodent studies (Brunson et al., 2005; Ivy et al., 2010) employed the water maze spatial memory test and identified deficits in both short- and long-term spatial memory as a consequence of exposure to early life unpredictability. The water maze may involve adverse conditions (swimming in water) and the stress of this task may moderate the link between early life unpredictability and memory performance, thus precluding the conclusion that unpredictability relates to memory function under non-stress conditions. Therefore, in more recent work we employed the novel object location and novel object recognition tasks, which do not involve a stressor component. The object location test probes hippocampus-dependent memory whereas the object recognition tasks interrogates multiple components of the limbic system. Performance deficits were observed on both tasks after early life exposure to unpredictability, and deficits were earlier and more severe in the more hippocampus- dependent object location test (Molet et al., 2016a; Davis et al., 2017; Short and Baram, 2019). In addition, rodent studies provide mechanistic insights: in rats, early life exposure to unpredictable sensory signals was associated with attenuated long-term potentiation, the cellular hallmark of memory, and with impoverished dendrites and synapses in dorsal hippocampus (Brunson et al., 2005; Ivy et al., 2010; Short and Baram, 2019), reflected in reduced dorsal hippocampus volumes (Molet et al., 2016a). These observed neuroanatomical consequences of early life exposure to unpredictability associate with observed performance deficits (Molet et al., 2016a).

### D. Summary

In summary, these cross-species studies highlight that unpredictable patterns of parental signals during the sensitive period of infancy may contribute to subsequent memory function and suggest that the developmental importance of unpredictable signals is conserved across species. Evidence suggests that unpredictable patterns of signals early in life may sculpt the developing brain, particularly neural circuits important for cognitive function. As discussed here, our recent research with humans indicates that unpredictable patterns of sensory signals in infancy sculpt corticolimbic circuit maturation in ways that partially mediate memory performance (Granger et al., 2021). Further, experimental and mechanistic research with rodents identify that the predictability of sensory input early in life may be a biological parameter influencing hippocampal circuit maturation (Birnie and Baram, 2022). Future directions in this research will probe the sex-specific consequences of early life exposure to unpredictable parental signals. While emerging research indicates that both males and females are impacted by unpredictability early in life, the functions that are vulnerable likely differ by sex (Glynn et al., 2018; Demaestri et al., 2020; Granger et al., 2021; Levis et al., 2021, 2022).

## IV Clinical implications and next steps

Our program of research, across laboratories and species, illustrates that exposure to early life adversity has profound and long-lasting implications for health and wellbeing. We have identified that early life exposure to patterns of parental sensory signals directly impact the developing offspring’s learning/memory process. This cross-species evidence supports the biological plausibility that patterns of sensory information from the parent during infancy are critical sources of input for the developing brain and that it is an evolutionarily conserved mechanism by which early life experiences shape neurodevelopment. The ability to test causality and mechanisms in pre-clinical experimental work with rats and mice in parallel with observational longitudinal research in humans and monkeys provides compelling support for the hypothesis that patterns of sensory signals during sensitive periods shape the development of neural circuits underlying cognitive function as well as those involved in sensory processing. Future work will continue to explore links between early life unpredictability and outcomes related to emotional development (Glynn et al., 2018), as well as implications for mental health later in life.

In line with recent calls for the examination of specific components of early life adversity (Boyce and Hertzman, 2018; Luby et al., 2020; Gee, 2021; McLaughlin et al., 2021), our findings suggest that unpredictable parental signals are a potent form of early adversity that shapes neurodevelopment cross species. Based on consequences of exposure to unpredictable signals, there is a need for clinically feasible screening tools to assess early life unpredictability in contexts where intensive coding is not feasible. To this end, we have developed and validated the Questionnaire of Unpredictability in Childhood (QUIC), (Glynn et al., 2019) which assesses unpredictable early experiences in the parenting and home environment before the age of 18 years. A full (38-item) and brief (5-item) version are available and validated in both English and Spanish (Glynn et al., 2019; Lindert et al., in press). Early screening is particularly important as unpredictability is a form of early adversity that can be addressed with efforts toward prevention and intervention. For example, we recently have shown that maintenance of family routines was protective during the acute responses to the COVID-19 pandemic and related shutdowns involving closure of schools and many other in-person activities. Importantly, maintenance of predictable family routines mitigated the negative impact of the pandemic on mental health outcomes among preschool aged children (Glynn et al., 2021) and was subsequently shown to be similarly protective for older children and adolescents (Rosen et al., 2021). This suggests that unpredictability is an actionable form of early life adversity that may be amenable to prevention efforts aimed at increasing predictability within the early life environment.

## Author contributions

EPD, TZB, MS, HSS, and KM conceptualized the review and drafted the sections of the manuscript. EPD, LMG, and CAS designed and conducted the human research. KM, DS, JB, and MS designed and conducted the monkey research. TZB and AKS designed and conducted the rodent research. HA and HSS performed the computations of entropy rate for all species and supported data analysis with EPD, AKS, and KM. All authors provided critical editorial feedback.

## Footnotes

1
https://contecenter.uci.edu/
