# Supplementary material for: Early life exposure to unpredictable parental sensory signals shapes cognitive development across three species
Source: Front Behav Neurosci. 2022 Oct 20;16:960262. doi: 10.3389/fnbeh.2022.960262 (PMC9630745; doi:10.3389/fnbeh.2022.960262)
Supplement: Supplementary file 1 [file Data_Sheet_1.docx]

**Supplement**

**Technical Details for Child Cognitive assessment:** Cognitive testing was performed when the child was 6.5 years using the Continuous Recognition Memory task (CRMT; Supplement Figure 1) which measures both memory encoding and retrieval. This task, assessing visual object recognition memory, has previously been shown to engage medial temporal regions such as the hippocampus [1] to be affected by early life adversity [2]. In the CRMT, children were presented with a series of 3 dimensional visual stimuli. Stimuli are colorful images presented on a black background. Two types of stimuli are presented. Concrete images, ones that can easily be named such as a banana and abstract images, 3D colorful objects without a clear name. 120 images were used, 60 concrete and 60 abstract. Prior to the task, children were given a practice round, using images not in the actual task, to ensure task comprehension. Because the Abstract trials were too challenging for young children, analyses focused only on concrete trials. All procedures were approved by the Institutional Review Board for the Protection of Human Subjects at the University of California Irvine and mothers provided written and informed consent for themselves and their infant.

**
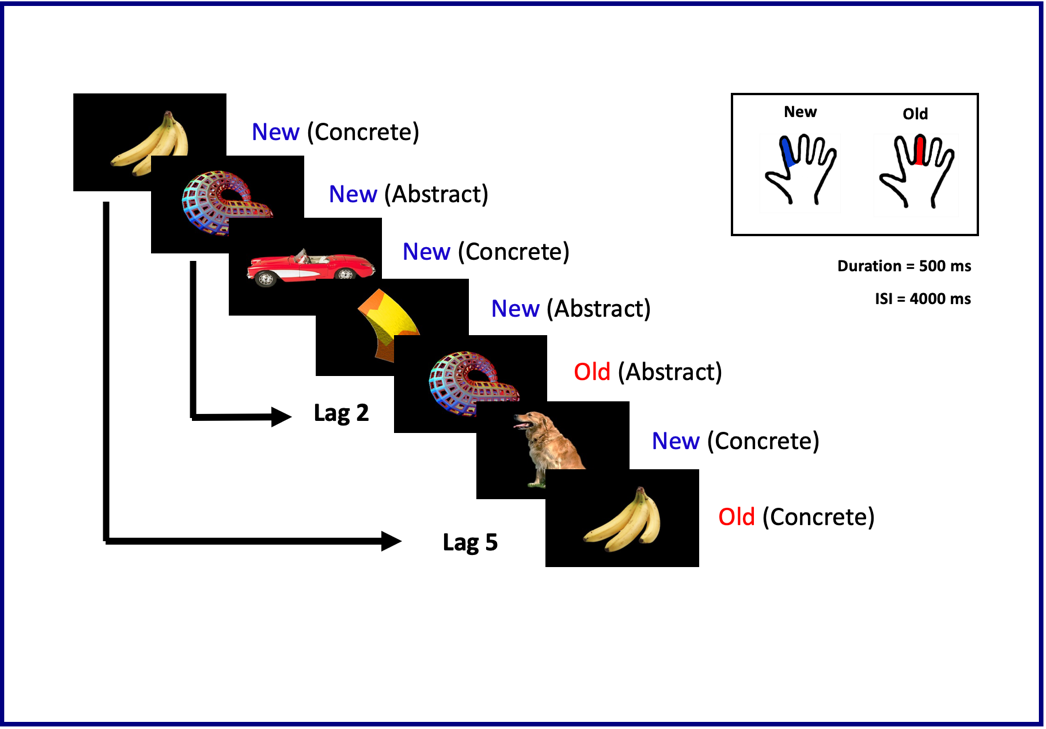
**

**Supplement figure 1**. Continuous Recognition Memory Task: In this task, every stimulus requires both memory retrieval (i.e., evaluation of the current stimulus against existing memory) as well as updating or encoding (i.e., noting that the image has repeated or that a new stimulus should be encoded).

**Technical details regarding Monkey cognitive assessment:** Cognitive tests were conducted using a Wisconsin General Testing Aparattus (WGTA) after subjects reached 18 months of age, roughly equivalent to school-age children (5-6 years). Before assessing working memory with the DNMS Session Unique task (DNMS-SU), all animals had to first reach criterion on the DNMS trial unique (DNMS-TU), which is a recognition memory task (Figure 2). The testing tray for the DNMS-TU contained three recessed wells for placement of rewards. The DNMS-TU task included a ‘familiarization’ phase during which a novel object called the *model object* was placed over the middle well, covering a food reward, the screen was raised, and the monkey was allowed to push the *model object* to retrieve the reward. Following a 10-second delay the screen was lifted to present the animal with the ‘choice’ phase, where the *model object* was placed on either the left or right lateral well and a novel object placed on the opposite lateral well, covering the reward. In order to correctly complete the trial, the subject had to displace the novel object. Each monkey was administered 30 trials of DNMS-TU each testing day, with two different objects presented per trial. Criterion was defined as 90% correct consecutive trials. After reaching criterion for DNMS-TU, monkeys were tested on the DNMS-SU [3], which is similar to DNMS-TU, but the same pair of objects is used across all 30 daily trials (each day is a session) and only changes across days/sessions. Thus, starting with the second trial and for all remaining session trials, both objects were seen and baited such that successful performance required the animal to remember the object he had seen most recently, testing its working memory. The following day a new pair of objects was used and so on until learning criterion was reached (90% correct choices).


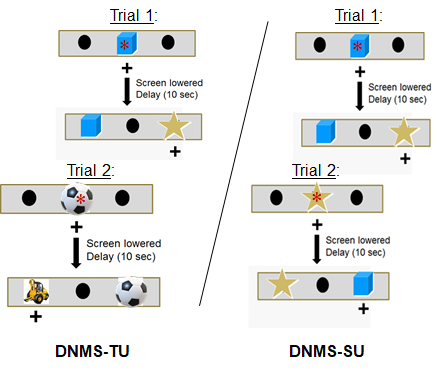


**Supplement figure 2.** Delayed Non-Match to Sample Task: Juvenile rhesus monkeys were tested in the delayed non-matching to sample- (DNMS) trial unique (DNMS-TU) task (Left), followed by the DNMS-session unique (DNMS-SU) task (Right). Red asterisk: model object; +: indicates baited well (containing food reward).

**Technical details regarding the calculations of entropy rates:** To better describe how entropy rate characterizes unpredictability, we briefly review the technical details that underlie the approach. As a starting point consider a randomly observed behavior X that is known to correspond to one of *k* states with $p_{i}$ as the probability of observing the behavior denoted as state *i*. One way of characterizing the uncertainty or unpredictability of such a random observation is through the entropy of the probability distribution. This is usually denoted by H and is computed as:


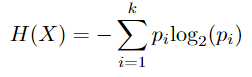


In our approach to understanding maternal behavior we focus not on individual behaviors but on the sequences. Thus, we consider a sequence of observed behaviors and model them as a random process known as a first-order Markov chain. A first order Markov chain is completely characterized by the transition matrix P whose elements P_ij_ specify the probability of a given row behavior (state i) being followed by a given column behavior (state j). Under fairly general conditions, if such a process was observed for a long period of time, there would be a consistent distribution of observed states known as the stationary distribution where π_i_ is the stationary probability or long-run frequency of the system being in state *i.* The entropy rate of the sequence of behaviors is calculated from the stationary distribution and the transition probabilities as :


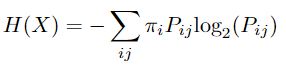


which is essentially a weighted average of the entropy for the distribution contained in each row of the transition matrix. Additional details regarding entropy rate and the approaches to calculating it can be found in Vegetabile et al. [4]. Higher entropy values indicate more unpredictability in the system. Entropy is a continuous measure that ranges between 0 to ${log}_{2}N$ where N is the number of states to which one can transition. This is generally one less than the number of states because we don’t allow for transitions from an observed behavior to another instance of the same behavior.

**References**

[1] H. Eichenbaum, A.P. Yonelinas, and C. Ranganath, The medial temporal lobe and recognition memory. Annu. Rev. Neurosci. 30 (2007) 123-152.

[2] O. Evren Güler, C.E. Hostinar, K.A. Frenn, C.A. Nelson, M.R. Gunnar, and K.M. Thomas, Electrophysiological evidence of altered memory processing in children experiencing early deprivation. Developmental science 15 (2012) 345-358.

[3] E. Heuer, and J. Bachevalier, Neonatal hippocampal lesions in rhesus macaques alter the monitoring, but not maintenance, of information in working memory. Behavioral neuroscience 125 (2011) 859.

[4] B. Vegetabile, S.A. Stout-Oswald, E.P. Davis, T.Z. Baram, and H. Stern, Estimating the entropy rate of finite markov chains with application to behavior studies. Journal of Educational and Behavioral Statistics 44 (2019) 282–308.
